# Supplementary material for: Molecular epidemiology of vancomycin-resistant Enterococcus faecium clinical isolates in a tertiary care hospital in southern Thailand: a retrospective study
Source: PeerJ. 2021 May 20;9:e11478. doi: 10.7717/peerj.11478 (PMC8141282; doi:10.7717/peerj.11478)
Supplement: Supplemental Information 1 [file peerj-09-11478-s001.docx]

Request accession Number for my manuscript

1. **Enterococcus faecium clinical strain S47_vanA**

**sequence F;**

ACGCTTCCTTCGCCGGATAATATGCACGGATTACTTGTTAAAAGGAACCA

TGAATATGAAATCAACCATGTTGATGTAGCATTTTCAGCTTTGCATGGCA

AGTCAGGTGAAGATGGATCCATACAAGGTCTGTTTGAATTGTCCGGTATC

CCTTTTGTAGGCTGCGATATTCAAAGCTCAGCAATTTGTATGGACAAATC

GTTGACATACATCGTTGCGAAAAATGCTGGGATAGCTACTCCCGCCTTTT

GGGTTATTAATAAAGATGATAGGCCGGTGGCAGCTACGTTTACCTATCCT

GTTTTTGTTAAGCCGGCGCGTTCAGGCTCATCCTTCGGTGTGAAAAAAGT

CAATAGCGCGGACGAATTGGACTACGCAATTGAATCGGCAAGACAATATG

ACAGCAAAATCTTAATTGAGCAGGCTGTTTCGGGCTGTGAGGTCGGTTGT

GCGGTATTGGGAAACAGTGCCGCGTTAGCTGTTGGCGAGGTGGACCAAAT

CAGGCTGCAGTACGGAATCTTTCGTATTCATCAGGAAGTCGAGCCGGAAA

AAGGCTCTGAAAACGCAGTTATAACCGTTCCCGCAGACCTTTCAGCAGAT

GAGCGAGGACGGATACCGGACACGGCAAATTCCATACATAAAATAACTCG

GCTGTACAGGTCTATTCTGTGTCAATATGTTTTTACTCGACAACGGACCA

AATGTAACAAAAAAAAAAAACATCCTTAGCTCTACCCAGACCTCAGCTCA

ATCACCAACTCTTCCTACTTATAAATATATAATATATCACCCTACCAACC

CTATCCCCTCA

**sequence R;**

TAAGGCTAACGGGCTAGACCTCTACAGCCGAGCGCTTTATATATTTTTTT

TGCCGTTTCCTGTATCCGTCCTCGCTCCTCTGCTGAAAGGTCTGCGGGAA

CGGTTATAACTGCGTTTTCAGAGCCTTTTTCCGGCTCGACTTCCTGATGA

ATACGAAAGATTCCGTACTGCAGCCTGATTTGGTCCACCTCGCCAACAGC

TAACGCGGCACTGTTTCCCAATACCGCACAACCGACCTCACAGCCCGAAA

CAGCCTGCTCAATTAAGATTTTGCTGTCATATTGTCTTGCCGATTCAATT

GCGTAGTCCAATTCGTCCGCGCTATTGACTTTTTTCACACCGAAGGATGA

GCCTGAACGCGCCGGCTTAACAAAAACAGGATAGGTAAACGTAGCTGCCA

CCGGCCTATCATCTTTATTAATAACCCAAAAGGCGGGAGTAGCTATCCCA

GCATTTTTCGCAACGATGTATGTCAACGATTTGTCCATACAAATTGCTGA

GCTTTGAATATCGCAGCCTACAAAAGGGATACCGGACAATTCAAACAGAC

CTTGTATGGATCCATCTTCACCTGACTTGCCATGCAAAGCTGAAAATGCT

ACATCAACATGGTTGATTTCATATTCATGGTTCTTTTTAACAAGTAATCC

GTGCATTTTTTTATCCGGCGAGAGTACAGCTGAATAGCAATTGCGGTTTT

CCCAAAAAAGAAGTGAGTGGAGCCCTCCCGAGCCCGAA

1. **Enterococcus faecium clinical strain S44_esp**

**sequence F;**

TTCTCACGGTTTGTTTATCAACCGCTTTTGGTGATTCCTTAATAACGGTT

GAACCTTCTTCTGGTTTATCAAAACCTGGAGAAACGATTTGGATTTTTAA

TTCATCTTTCGCGATTAATTTACTTGAATCTACGCCCGAAAATTCAAGTT

CTACAGTTGGATTAATACACCAACAACGGACTGACAAAAAACAAACCATC

TGGCAGCACCAAACTACCTCCCAGAGCCCGAACTGAGTCCCGTGAGCGTC

TACTCAATTCCCCGTGCACTGTTGTCCAGGGTGTTCGCCTATTGGTCCTA

CTTGCGGATTCTCTTTCCTCCCTTATTCTCCCCCGGTTGTGCCAATGTGT

GTT

**sequence R;**

GCCGGGTCGTGTCAATTAGTCCAGAACACTTATGGAACAGGTTATTATTA

TTTGCAAGATATTGATGGTGATGGACCTCTGACGATACTACCTGCCATGC

ACGATAGTGCCCACGAGCAATGCCCCATCCGGTTTGGCACCAGATCCACC

TTCTGGGAAAACATGGAGTGATCAGCCACCGGATGACTGACACAAGACAG

AGCATCTGGCTTCTTGAGAGCGCCGTTCCCGAATCGGACATGTGGCCCTG

TATTTTCCTTCCCCAATCTGCCCAGACTTGCGGCCTGGTTGTTCGCCTTT

TGGGTCCTCTTCTGGTCCCTCTCTCACTTACTCATTGCTCAAGATCGTGC

GCCTGTGGCTAAGGTTATCTTCTCATTCGAAGGGGGTAATTTTTTAA

1. **Enterococcus faecium clinical strain 467-1_hyl**

**sequence F;**

CGTCTCCGGCCGATGCTGATTTGGGATAATATTCCGGTAAATGATTATTT AGAGGATAAAGAACTATTGTTCATGAGTCCATATGAGAATCGAACACCGA ATCTTTCTAAGGAGAGATACCAGGTTACAGGAGTATCTTCGTCTCCAATG GCTATATTATACGCTTAAACGATACCGCTACAGTCGGGGTTTTGGGGGGA CGTGAGGAAACTCCGTTCTGGATAGGGGATCACAGGTACACTTGGCACCT GAACGGGAAGGGGCAGGTAGGACAGGGTAGCCGGTACACCTGGGTTCCAG CGAGGGGTGTTGATGAAGATGCGGTTGCGGAACCGATCAACGCCCACCGG GAGGTTGTTCTCCGGAATGACCCTATATGATTTTTTGATGTAATA

**sequence R;**

CGTCTCCGGCCGATGCTGATTTGGGATAATATTCCGGTAAATGATTATTT AGAGGATAAAGAACTATTGTTCATGAGTCCATATGAGAATCGAACACCGA ATCTTTCTAAGGAGAGATACCAGGTTACAGGAGTATCTTCGTCTCCAATG GCTATATTATACGCTTAAACGATACCGCTACAGTCGGGGTTTTGGGGGGA CGTGAGGAAACTCCGTTCTGGATAGGGGATCACAGGTACACTTGGCACCT GAACGGGAAGGGGCAGGTAGGACAGGGTAGCCGGTACACCTGGGTTCCAG CGAGGGGTGTTGATGAAGATGCGGTTGCGGAACCGATCAACGCCCACCGG GAGGTTGTTCTCCGGAATGACCCTATATGATTTTTTGATGTAATA
